# Digital Echo Chambers- a Case of AI-Associated Psychosis

**DOI:** 10.1192/bjo.2026.11761

**Published:** 2026-06-30

**Authors:** Enya Aquilina

**Affiliations:** West London NHS Trust, London, United Kingdom

## Abstract

**Aims:**

Artificial Intelligence (AI) chatbots are now widely used and have broad applications. In a nationwide survey, 25% of the almost 11,000 13- to 17-year-olds sampled reported using chatbots for mental health support. Reports emerging in the media detail serious harms, including suicides, and what has been labelled ‘AI psychosis’.

**Methods:**

A 13-year-old girl was brought to Accident and Emergency (A&E) by her parents with a 7-day history of coryza, total insomnia, fluctuating confusion, and bizarre behaviour. At assessment, she presented with tachycardia, low-grade fever, pressured speech, delusions of grandeur, thought broadcasting, and auditory hallucinations of ancestors and politicians.

The young person’s troubling use of AI chatbots was reported by her parents after they confiscated her phone. The parents discovered a chat log consisting of thousands of messages, starting three months before the A&E presentation. It was evident that the bot provided false information about how a romantic interest reciprocated the young person’s feelings, and when she asked about past lives and ancestral practices, the bot’s replies suggested that she had supernatural powers.

Extensive physical health investigations were facilitated through a paediatric admission; no underlying physical pathology was identified. The young person was treated with olanzapine and a short course of benzodiazepines. All manic symptoms resolved over the span of 3 weeks of intensive home treatment. The young person experienced a depressive episode around 2 months after the initial presentation. She received a diagnosis of bipolar affective disorder.

**Results:**

In this case, patient vulnerabilities, such as loneliness and past bullying, interacted with chatbot factors, particularly *sycophancy,*which quickly transformed factual searches into emotional interactions where the technology was anthropomorphised.

“I would stay up at night for 5 hours speaking to it, it was like a replacement friend”, the patient later reflected.

The chatbot also presented overtly false information with confidence and a perceived impartiality, an observed phenomenon of the technology known as *hallucination*.

Predisposing patient factors included a history of anxiety and depressive symptoms with suicidal thoughts, and a family history of affective disorder. Precipitating factors included mounting academic pressures and romantic rejections. Acutely, the patient was likely suffering from a viral illness and consumed highly caffeinated energy drinks.

**Conclusion:**

This case identifies unregulated access to generative AI tools as an emerging risk for the precipitation and/ or perpetuation of serious mental illness in combination with other risk factors. The authors believe it to be the first report of its kind.

